# Validation and reliability study of the Turkish version of the Neuroquality of Life (Neuro-QoL)-Stigma Scale for neurological disorders

**DOI:** 10.3906/sag-1811-50

**Published:** 2019-06-18

**Authors:** Sibel KARŞIDAĞ, Nilgün ÇINAR, Şevki ŞAHİN, Nurdan KOTEVOĞLU, Miruna Florentina ATEŞ

**Affiliations:** 1 Department of Neurology, Faculty of Medicine, Maltepe University, İstanbul Turkey; 2 Department of Physical Therapy and Rehabilitation, Faculty of Medicine, Maltepe University, İstanbul Turkey

**Keywords:** Stigma, neurology, Neuro-QoL, validation

## Abstract

**Background/aim:**

Stigma can be defined as a negative perception of chronically ill patients by their relatives or by society, or a similar self-perception by the patients themselves. We aimed to validate the Turkish version of the Neuroquality of Life (Neuro-QoL)-Stigma Scale for neurologic diseases.

**Materials and methods:**

Forms were filled out by a total of 152 randomized patients under regular follow-up in the outpatient clinic (29 polyneuropathy, 25 epilepsy, 23 stroke, 24 tension-type headache, 28 multiple sclerosis, 27 Parkinson disease). The forms consisted of the Beck Depression Inventory (BDI), Beck Anxiety Inventory (BAI), WHOQOL**-**BREF quality of life scale, the Multidimensional Scale of Perceived Social Support (MSPSS), the General Self-Efficacy (GSE) scale, and the Neuro-QoL-Stigma scale.

**Results:**

The internal consistency of the Neuro-QoL-Stigma scale showed Cronbach’s α coefficients of 0.95 for all groups. The mean scores of the stigma scales were 33.42 ± 13.91 (min–max: 24–87). There were strong negative correlations between high stigma scores and GSE-T, MSPSS-T, and WHOQOL-BREF, and a positive correlation with the BDI and BAI.

**Conclusion:**

The Turkish version of Neuro-QoL-Stigma has satisfactory content validity and high internal consistency. Neuro-QoL-Stigma is suitable for understanding stigmatization in different neurological disorders in the Turkish population. The scale is available for use at http://www.healthmeasures.net/explore-measurement-systems/neuro-qol.

## 1. Introduction

Stigmatization is the negative labeling of an individual or specific situation and this labeling results in the exclusion of the individual from society (1). Stigmatization worsens the quality of life (QoL), increases public prejudice, causes a reduction in the perception of self-worth, has negative physical and psychological consequences, and disrupts work and family life (2–4).****Stigma is a term used frequently in relation to mental disorders, but it is still uncommon to evaluate stigmatization in the context of neurological diseases (5). However, the local burden and cultural particulars of stigmatization should be further evaluated using cross-cultural comparisons.

The various forms of stigma manifest themselves as public stigma, self-stigma (internal), and label avoidance in the DSM-V. Stigma scales target these three areas (6). Assessment of stigmatization is very important because stigmatization itself may result in delay in treatment choice and decrease in treatment quality. Stigmatization in neurological disorders has a severe effect on the patient’s family and social relationships (7,8).

In Turkey, there is a limited number of studies that evaluate stigmatization in neurological disorders such as epilepsy, tension-type headache, and Parkinson disease, and there is no scale used specifically for targeting neurological disorders (9–12). Neuro-QoL is a scale developed by the National Institute of Neurological Disorders and Stroke. Neuro-QoL consists of 13 adult and 10 pediatric subgroup tests including physical, mental, and social health (13). It is a health assessment tool that is clinically and psychometrically appropriate for major neurological disorders (i.e. stroke, epilepsy, multiple sclerosis, amyotrophic lateral sclerosis, and Parkinson disease) (14,15). 

The stigma scale is one of the subscales of Neuro-QoL for pediatrics and adults in both the hard copy and computerized version. The adult long version consists of 24 items and the short version consists of the first 8 items of the long version (13). ****

Our aim was to evaluate the reliability and validity of the adult long version of the Neuro-QoL-Stigma scale in order to use it in clinical practice and research among Turkish patients. 

## 2. Materials and methods

### 2.1. Study design

The present study was established in two stages. The first stage was the cross-cultural adaptation. A sequential approach was followed in order to obtain linguistically equivalent versions of the adult long version of the Neuro-QoL stigma scale consisting of 24 items. Semantic equivalence was achieved with two independent translations into Turkish performed by two bilingual Turkish experts and a consensus version, followed by a final back-translation performed by another translator whose native language was English and who was also fluent in Turkish.

The quality analysis of the translations and the first content validity were checked by a clinician’s review and by a cognitive debriefing panel with 10 healthy literate people. After completing the questionnaires, we asked them to explain the complicated and difficult issues to the participants. Corrections were made according to suggestions deemed suitable.

In the second phase, the reliability and validity tests of the Turkish version were examined. We evaluated the internal consistency reliability and construct validity of the stigma subscales of Neuro-QoL. Internal consistency reliability was evaluated by examining the item-total correlations and Cronbach’s alpha coefficients. The item-total correlations were calculated by removing each of the 24 items. We calculated and reported the alphas when any one of the items was removed from the instrument. Cronbach’s alpha was also reported for the whole instrument. For Cronbach’s alpha, we considered the following cut-off values: >0.7 (acceptable), >0.8 (good), and >0.9 (excellent). For item-total correlation, we considered a value greater than 0.3 to be an indicator that an item was related to the overall scale. Concurrent validity was tested by comparing other related scales. Stigma scores were calculated as a t-scores (50 is the mean and 10 is the standard deviation) (16).

### 2.2. Patients

Patients with neurological diseases were consecutively recruited from the outpatient clinic of our neurology department. Six major neurological diseases were identified: relapsing-remitting multiple sclerosis (MS), Parkinson disease (PD), ischemic stroke, tension-type headache, generalized epilepsy, and chronic polyneuropathy. Literate patients were preidentified in terms of compliance to the test and those who scored 27 or higher on the Turkish version of the Mini Mental State Examination (MMSE-T) (17) were enrolled in the study. Sociodemographics and clinical characteristics including age, sex, education, employment status, marital status, and hospitalization were assessed.

### 2.3. Instruments

The Neuro-QoL stigma subscale consists of 24 items. Item scores range from 1 (never) to 5 (always). A summary index is calculated by adding all scores, ranging from 24 to 120, with higher scores reflecting the worst stigmatization (13).

The World Health Organization’s Quality of Life (WHOQOL)-BREF with 26 items is a generic measure of health status comprising four major health dimensions (physical health, psychological health, social relationships, environment), all of them on a scale of 26–135, with higher scores indicating full health status (18,19). In this study, the Turkish version of the WHOQOL-BREF was used (20).

The Beck Depression Inventory (BDI) with 21 items is one of the popular depression measures. Scores are in the range of 0–63, with higher scores indicating severe depression. In this study, the Turkish version of the BDI was used (21–23).

The Beck Anxiety Inventory (BAI) with 21 items is a severity indicator for anxiety. Scores are in the range of 0–63, with higher scores indicating severe anxiety (24). In this study, the Turkish version of the BAI was used (25).

The General Self-Efficacy (GSE) scale measures the belief in one’s ability to complete activities related to one’s own competency. The GSE contains 17 items, 6 of which assess the level of positive self-esteem and 11 of which evaluate the level of negative self-esteem. Each item is scored from 1 (completely disagree) to 5 (completely agree) (26). In this study, the Turkish version of the GSE was used (27).

The Multidimensional Scale of Perceived Social Support (MSPSS) is a research tool that assesses social support. It consists of three subscales, each of them representing a different source of support: family, friends, and significant other. It contains 12 items rated on a seven-point Likert-type scale with scores ranging from ‘very strongly disagree’ (= 1) to ‘very strongly agree’ (= 7) (28). In this study, the Turkish version of the MSPSS was used (29).

### 2.4. Statistical analysis

Descriptive statistics were applied to demographic and questionnaire data. Internal consistency reliability was tested by Cronbach’s alpha indicator. Scores between 0.70 and 0.95 are considered as acceptable reliability indicators. Concurrent validity was tested by comparing the measured scores (one-way ANOVA with Tukey post hoc test for pairwise comparisons) among known group variables as well as by Pearson’s correlation coefficient. Pearson’s correlation coefficients were computed following the same criteria as above. P < 0.05 indicated statistical significance.

### 2.5. Ethics 

Information about the research was given to all participants. The study protocol and ethics procedures were approved by the ethics board of our institution. Patients or their legal guardians provided informed signed consent.

## 3. Results 

From a total of 152 patients, 65% were female and 35% were male. The mean age was 47.9 ± 17.6 years. The study group consisted of 29 patients with polyneuropathy, 25 patients with epilepsy, 23 patients with stroke, 24 patients with tension-type headache, 28 patients with MS, and 27 patients with PD.

Table 1 shows the mean scores, the standard deviations, internal consistency correlations (ICCs), and Cronbach’s **α **results for each domain of the Neuro-QoL-Stigma scale. Table 2 shows the correlation matrix obtained and the significance values. Comparing Neuro-QoL-Stigma and other general measures, we found strong negative correlations with the GSE, MSPSS, and WHOQOL-BREF-T. We also found strong positive correlations with the BDI and BAI. We found no significant correlation with the family subdimension of the MSPSS. 

**Table 1:** Mean values and confidence intervals of reliability analysis for Neuro-QoL-Stigma scale. Variables presented as mean ± standard deviation (SD) because of normal distribution. Cut-off values of Cronbach’s alpha are as follows: >0.78 (acceptable), >0.8 (good), and >0.9 (excellent). Internal consistency correlations (ICCs) are statistically significant at P ≤ 0.001.

Item no.	Item	Mean ± SD	Cronbach’s α	ICC
1	Because of my illness, some people avoided me	1.32 ± 0.84	0.945	0.70
2	Because of my illness, I felt left out of things	1.30 ± 0.84	0.944	0.82
3	Because of my illness, people avoided looking at me	1.26 ± 0.74	0.945	0.74
4	I felt embarrassed about my illness	1.39 ± 0.90	0.945	0.73
5	Because of my illness, some people seemed uncomfortable with me	1.50 ± 0.90	0.945	0.70
6	I felt embarrassed because of my physical limitations	1.32 ± 0.79	0.944	0.77
7	Because of my illness, people were unkind to me	1.11 ± 0.37	0.947	0.67
8	Some people acted as though it was my fault I have this illness	1.30 ± 0.67	0.948	0.39
9	Because of my illness, I felt embarrassed in social situations	1.38 ± 0.94	0.944	0.78
10	Because of my illness, I felt emotionally distant from other people	1.60 ± 1.05	0.943	0.81
11	Because of my illness, people tended to ignore my good points	1.30 ± 0.71	0.947	0.59
12	Because of my illness, I was treated unfairly by others	1.24 ± 0.68	0.947	0.54
13	Because of my illness, I felt different from others	1.63 ± 1.04	0.943	0.82
14	Because of my illness, I worried about other people’s attitudes towards me	1.49 ± 1.01	0.944	0.79
15	Because of my illness, I worried that I was a burden to other	1.92 ± 1.19	0.945	0.71
16	Because of my illness, people made fun of me	1.08 ± 0.29	0.949	0.34
17	I was unhappy about how my illness affected my appearance	1.81 ± 1.25	0.948	0.60
18	Because of my illness, strangers tended to stare at me	1.32 ± 0.77	0.946	0.65
19	I lost friends by telling them that I have this illness	1.13 ± 0.49	0.948	0.42
20	Because of my illness, it was hard for me to stay neat and clean	1.46 ± 0.96	0.946	0.62
21	I felt embarrassed about my speech	1.30 ± 0.80	0.946	0.62
22	I avoided making new friends to avoid telling others about my illness	1.33 ± 0.87	0.945	0.69
23	I tended to blame myself for my problems	1.48 ± 0.92	0.946	0.66
24	People with my illness lost their jobs when their employers found out about it	1.42 ± 0.89	0.950	0.33

**Table 2:** Correlation between Neuro-QoL-Stigma and the other generic measures.

The names of other generic measures	Neuro-QoL-Stigma (P)
GSE	–0.30**
MSPSS	–0.38**
MSPSS Family	–0.10
MSPSS Friends	–0.42**
MSPSS Significant Other	–0.34**
BDI	0.56**
BAI	0.46**
WHOQOL-BREF-T, physical health	–0.38**
WHOQOL-BREF, psychological health	–0.55**
WHOQOL-BREF, social relationships	–0.46**
WHOQOL-BREF, environment	–0.31**

GSE: General Self-Efficacy, MSPSS: Multidimensional Scale of Perceived Social Support, BDI: Beck Depression Inventory, BAI: Beck Anxiety Inventory, WHOQOL-BREF: World Health Organization Quality of Life-BREF, *P < 0.05, **P < 0.01.

T-scores of the Neuro-QoL-Stigma scale for each group were 48.7 ± 11 for polyneuropathy, 49.1 ± 4.9 for epilepsy, 50.4 ± 11 for stroke, 45.2 ± 2.8 for headache, 51 ± 10.3 for MS, and 55.2 ± 12.5 for PD. When comparing our patients’ Neuro-QoL stigma T-scores with mean clinical T-scores of the Neuro-QoL study reference group (national), score difference of less than 0.5 SD units were determined within normal limits. When the groups were compared according to T-score, there was a significant difference between the headache and the PD group (P = 0.03).

## 4. Discussion

Stigma can be defined as a negative perception of chronically ill patients by their relatives or by society, or a similar self-perception by the patients themselves. Patients try to hide their illness from other people because of their feelings of embarrassment (30).

Stigma is usually associated with neurological diseases as well as psychiatric illnesses so it is important to use a scale that can evaluate stigma for neurologists. With neurological diseases, stigma can lead to anxiety, depression, and decreased self-esteem and diminished life satisfaction. With the addition of long-term health problems, quality of life can be negatively affected (31). Our results showed a strong correlation with the scales (GSE, BDI, BAI, MSPSS, and WHOQOL-BREF-T) in support of the above information. 

Rao et al. developed a stigma scale for chronic disease. They described three types of stigmatization as follows: perceived stigma (discriminatory attitude by society), enacted stigma (experience of social prejudices), and self-stigmatization (internalization of negative behaviors and low self-esteem) (7). Neuro-QoL Stigma also contains such areas.

Various factors have been indicated in stigma studies in neurological diseases. In a stigma study performed among epilepsy patients, educational status, level of income, age at onset of the disease, and frequency of seizures were shown as influencing factors. Having adequate social support and increased self-efficacy has been found to have a positive effect on stigma scores. Increased knowledge of patients about epilepsy and the presence of positive attitudes towards epilepsy were associated with decreased stigma scores in patients with epilepsy (32,33). Victorson et al. reported that the T-score was 49.7 ± 9.1 in adult epilepsy patients by using the Neuro-QoL stigma scale (34). In the Neuro-QoL user manual, the T-score was defined as 50.6 ± 6.7 for epilepsy (16). We found the T-score for epilepsy to be 49.1 ± 4.9. This result is consistent with previous studies.

PD patients have feelings of shame related to their movement and communication difficulties. Patients who cannot cope with social life due to their symptoms and who withdraw from society will have to live alone in their private world. Studies showed that higher stigma scores were related to more severe PD symptoms (35). Nowinski et al. showed that the stigma T-score was 48.39 ± 6.62 in PD cases (36). The Neuro-QoL user manual described the T-score as 49.29 ± 4.65 in PD (16). We found that the T-score for PD was 55.2 ± 12.5. Our results are higher than those of previous studies. This may be due to the possibly high level of disability in our PD cases. Further studies are required.

MS is one of the most common causes of severe disability in young people. Apart from the neurological findings of MS, stigmatization, which affects the quality of life, must be considered by physicians. In stigma studies of MS, it was found that higher Expanded Disability Status Scale (EDSS) scores with higher age, longer disease duration, and progressive forms are found to be responsible for stigma (37,38). Miller et al. found average T-scores for MS cases as 49.3 ± 7.23 by using the Neuro-QoL short form (39). The Neuro-QoL user manual indicated the T-score as 50.13 ± 5.2 for MS (16). We found this score as 51 ± 10.3 for MS. 

Stroke-related stigma studies show that stroke survivors who experience mild-to-moderate levels of stigma are more likely to be depressed or have lower quality of life (40). The mean stigma T-score was 51.94 ± 6.33 for stroke patients in the Neuro-QoL user manual (16). We found the T-score as 50.4 ± 11 for stroke patients. 

It was found that tension-type headache is considerably more stigmatized than migraine in headache studies; patients may hide their symptoms and will not seek help or treatment (41). Young et al. investigated stigma in patients with episodic migraine, chronic migraine, and epilepsy. They observed that patients with chronic migraine and epilepsy had similar stigmatization. In their study, stigma correlated with inability to work (42). In the current study, we found the lowest T-score as 45.2 ± 2.8 in the headache group. Further studies are needed in different types of headache.

In some polyneuropathy studies, it is found that some new developments have decreased the burden of stigmatism on patients and families over the past few decades. This may be due to recent medical treatments, primary care health professionals, and ongoing clinical trials (43). We found that the T-score was 48.7 ± 11 for the polyneuropathy group. 

Stigma may vary according to cultural differences, education level, and many other social and clinical factors. If patients cannot accept or understand their diagnosis, they cannot develop insight regarding their illness. This is the most important cause of self-stigmatization of patients (12). 

Stigma should be studied since it often accompanies neurological diseases. We aimed to evaluate the reliability and validity of the stigma scale of the Neuro-QoL tool. This scale can be used in clinical practice among different neurological diseases to understand stigmatization and it can be effective in treatment planning and prognosis of neurological diseases. All parameters of the adult long form of the Neuro-QoL-Stigma scale demonstrated high internal consistency and it is suitable for the Turkish population.
